# Thiamethoxam-Induced Intergenerational Sublethal Effects on the Life History and Feeding Behavior of *Rhopalosiphum padi*

**DOI:** 10.3390/plants13060865

**Published:** 2024-03-17

**Authors:** Hina Gul, Ihsan ul Haq, Ali Güncan, Arzlan Abbas, Shanza Khan, Aqsa Yaseen, Farman Ullah, Nicolas Desneux, Xiaoxia Liu

**Affiliations:** 1 MARA Key Laboratory of Pest Monitoring and Green Management, Department of Entomology, College of Plant Protection, China Agricultural University, Beijing 100193, China; 2 Insect Pest Management Program, Institute of Plant and Environmental Protection, National Agricultural Research Centre, Islamabad 44000, Pakistan; 3 Department of Plant Protection, Faculty of Agriculture, Ordu University, 52200 Ordu, Turkey; guncan.ali@gmail.com; 4 College of Plant Protection, Jilin Agricultural University, Changchun 130118, China; zain514786@gmail.com; 5 State Key Laboratory for Managing Biotic and Chemical Threats to the Quality and Safety of Agro-Products, Institute of Plant Protection and Microbiology, Zhejiang Academy of Agricultural Sciences, Hangzhou 310021, China; farmanullah787@gmail.com; 6 Université Côte d’Azur, INRAE, CNRS, UMR ISA, 06000 Nice, France

**Keywords:** bird cherry-oat aphid, aphids, neonicotinoids, life table, electrical penetration graphs, ecotoxicology

## Abstract

Thiamethoxam, a second-generation neonicotinoid insecticide is widely used for controlling sap-sucking insect pests including *Rhopalosiphum padi*. The current study aimed to investigate the life-history parameters and feeding behavior of *R. padi* following treatments with sublethal concentrations of thiamethoxam. The lethal concentration 50 (LC_50_) value of thiamethoxam against adult *R. padi* was 11.458 mg L^−1^ after 48 h exposure. The sublethal concentrations of thiamethoxam (LC_5_ and LC_10_) significantly decreased the adult longevity, fecundity, and reproductive days in the directly exposed aphids (F_0_ generation). In the progeny *R. padi* (F_1_), the developmental durations and total prereproductive period (TPRP) were decreased while the adult longevity, fecundity, and reproductive days (RP*_d_*) were increased at both thiamethoxam concentrations. The demographic parameters including the net reproductive rate (*R*_0_), intrinsic rate of increase (*r*), and finite rate of increase (*λ*) were prolonged only at the LC_5_ of thiamethoxam. The EPG results indicated that the sublethal concentrations of thiamethoxam increases the total duration of non-probing (Np) while reducing the total duration of E2 in directly exposed aphids (F_0_). Interestingly, the E2 were significantly increased in the progeny generation (F_1_) descending from previously exposed parental aphids (F_0_). Overall, this study showed that thiamethoxam exhibited high toxicity against directly exposed individuals (F_0_), while inducing intergenerational hormetic effects on the progeny generation (F_1_) of *R. padi*. These findings provided crucial details about thiamethoxam-induced hormetic effects that might be useful in managing resurgences of this key pest.

## 1. Introduction

The bird cherry-oat aphid, *Rhopalosiphum padi* (Hemiptera: Aphididae), is a damaging sap-sucking insect that attacks cereal crops all over the world [1,2]. It can cause direct damage by feeding on cereal plants and indirect damage by serving as a vector for the spread of numerous viruses [3]. This pest causes severe economic damage to wheat crops throughout the world. Even though there were various management strategies [4,5,6], insecticides remained a major option for controlling insect pests [7,8,9]. However, biotic and abiotic factors might cause these insecticides to degrade in the field to sublethal and low lethal concentrations [10,11]. Exposure to these sublethal concentrations results in a variety of non-lethal effects on insect pests, such as hormesis, resistance development, physiology, and behavior changes, etc. [12,13,14].

Thiamethoxam, a potent neonicotinoid insecticide, effectively controls sap-sucking insect pests including aphids [15,16,17]. However, insects are exposed to sublethal and low lethal concentrations of chemical insecticides after degradation via abiotic and biotic constraints [11,12] that ultimately affect the life-history parameters as well as inducing resistance development in the directly exposed individuals and subsequent generations [18,19,20,21]. The demographic toxicology or life table approaches have been considered crucial for evaluating the impact of abiotic factors on biological traits of insect populations such as survival, reproduction, development, life expectancy and fecundity [22,23]. Life-history parameters act as indicators for evaluating sublethal effects [24]. Insecticide-induced lethal, sublethal, intergenerational/multigenerational, and hormesis effects on insect pests were comprehensively assessed using the age-stage, two-sex life table, integrating data from both (male and female) sexes for an accurate evaluation of these parameters [25,26,27]. Meanwhile, laboratory and field studies offer crucial insights into insecticide-induced mortality. However, additional strategies are required to obtain a comprehensive understanding of how a pesticide affects the feeding behavior of insects.

Electrical penetration graphs (EPGs) are the most precise method for determining an insecticide’s impact on plant tissue damage and feeding behavior of piercing-sucking insect pests [28,29,30]. The alteration in resistance and biopotentials (plant interior voltage) generates waveforms that correspond to insect-feeding activities, encompassing salivation, ingestion, stylet-induced cell puncturing, insertion, and position within plant tissue.

In this study, we established the sublethal concentrations (LC_5_ and LC_10_) by determining an acute toxicity response of thiamethoxan on the wheat aphid *R. padi*. Employing an age-stage, two-sex life table approach, we investigated the sublethal effects of thiamethoxam on the survival, fecundity, development, and population projection of *R. padi*. Furthermore, we used EPG to investigate the impact of sublethal concentrations of thiamethoxam on the feeding behavior of parental and progeny generations.

## 2. Results

### 2.1. Toxicity of Thiamethoxam against Rhopalosiphum padi

The toxicity of thiamethoxam against adult *R. padi* was investigated following 48 h exposure. Results showed that the LC_5_, LC_10_, and LC_50_ of thiamethoxam against adult *R. padi* were 2.844 mg L^−1^ (1.900–3.762), 3.869 mg L^−1^ (2.772–4.902), and 11.458 mg L^−1^ (9.766–13.416) with slope ± SE of 2.718 ± 0.283 (χ^2^ = 5.458, *df* = 13, *p* = 0.963) (Table S1).

### 2.2. Impact of Sublethal Concentrations of Thiamethoxam on Parental Rhopalosiphum padi (F_0_)

The F_0_ generation of *R. padi* exhibited substantial alterations in adult longevity, fecundity, and reproductive duration following exposure of the sublethal concentrations (LC_5_ and LC_10_) of thiamethoxam (Table 1). The *R. padi* adult exposed to the LC_5_ and LC_10_ of thiamethoxam for 48 h showed significant reduction (*p* < 0.05) in adult longevity compared to the control. The *R. padi* treated with sublethal concentrations (LC_5_ and LC_10_) of thiamethoxam had a decreased fecundity. Furthermore, exposure to the LC_10_ of thiamethoxam had the lowest number of reproductive days compared to LC_5_ and control aphids.

### 2.3. Developmental Duration and Adult Longevity of Rhopalosiphum padi Progeny Generation (F_1_)

The effects of sublethal concentrations of thiamethoxam on the developmental time and longevity of progeny generation *R. padi* (F_1_) are presented in Table 2. The results revealed a substantial reduction (*p* < 0.05) in the developmental duration of 1st, 3rd, and 4th instars at LC_5_ concentrations of thiamethoxam while no effects were observed for the LC_10_ group compared to the control (Table 2). The developmental duration of the 2nd instar was decreased at both concentrations. However, the pre-adult stage of *R. padi* (F_1_) was significantly decreased (*p* < 0.05) compared to the control group. Consequently, exposure to the LC_5_ and LC_10_ of thiamethoxam resulted in a considerable improvement (*p* < 0.05) in the adult longevity. However, the total longevity of female progeny aphids (F_1_) was significantly increased only at LC_5_ of thiamethoxam as compared to the control.

### 2.4. Demographic Traits and Fecundity of Rhopalosiphum padi Progeny Generation (F_1_)

The intergenerational effects of thiamethoxam (LC_5_ and LC_10_) on the fecundity and demographic parameters of progeny generations of *R. padi* (F_1_), whose parents (F_0_) were subjected to LC_5_ and LC_10_ concentrations, are depicted in Table 3. The net reproductive rate, *R*_0_ (offspring/individual), the intrinsic rate of increase *r* (day^−1^), and the finite rate of increase *λ* (day^−1^) significantly increased (*p* < 0.05) at LC_5_ concentrations of thiamethoxam, while no effects were observed on LC_10_ as compared to the control group. The fecundity (nymphs/female) and reproductive days (RP*_d_*) of F_1_ individuals was substantially increased (*p* < 0.05) at both the LC_5_ and LC_10_ concentrations of thiamethoxam (Table 3), while no significant effects (*p* > 0.05) were observed on the adult pre-reproductive period (APRP). However, the total pre-reproductive period (TPRP) was significantly decreased at both thiamethoxam concentrations, while the mean generation time (*T*) was reduced only at LC_5_ as compared to the control aphids.

**Figure 1:**
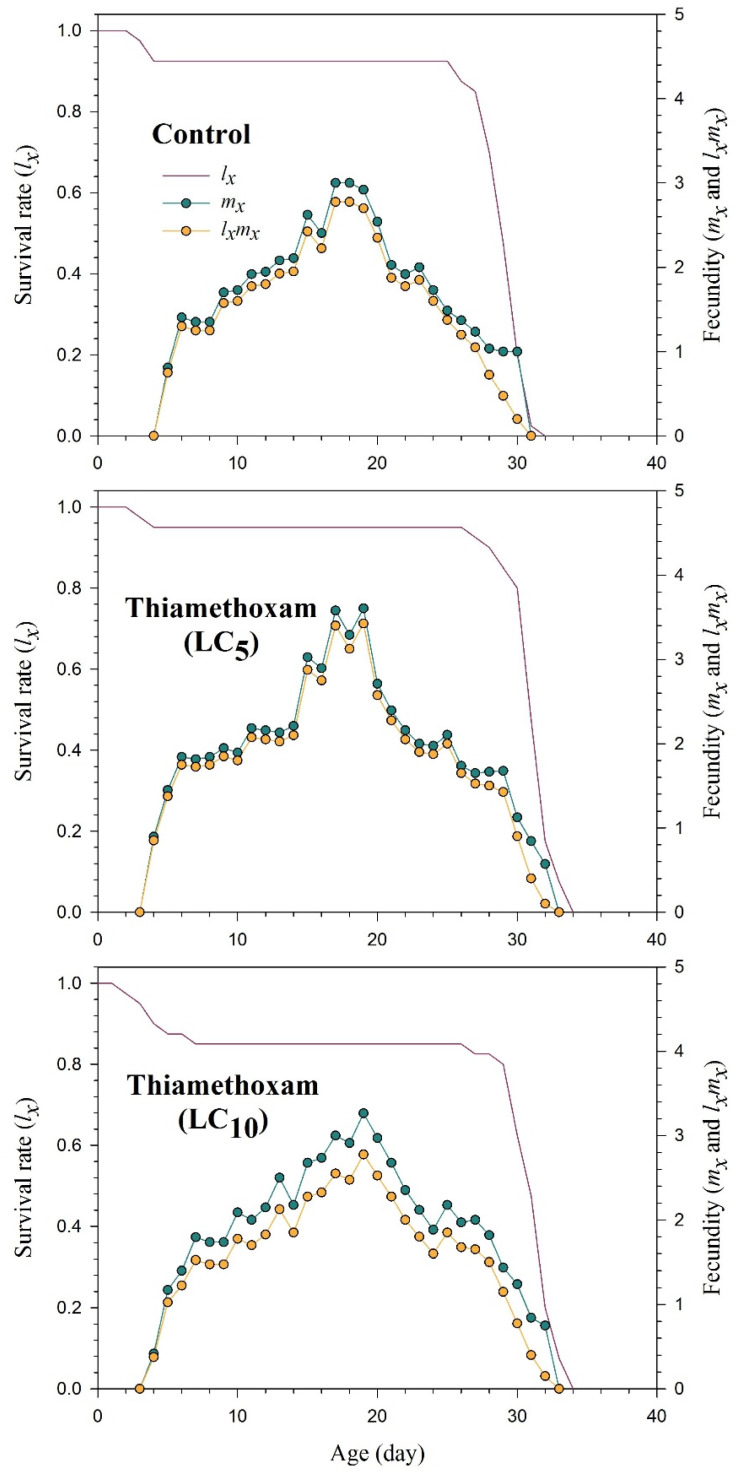
Age-specific survival rate (*l_x_*), age-specific fecundity (*m_x_*), and the age-specific maternity (*l_x_m_x_*) of progeny *Rhopalosiphum padi* (F_1_) produced from parental aphids exposed to the sublethal concentrations of thiamethoxam.

The curves in Figure S1 illustrated the probability of survival for newly born nymphs of *R. padi* to reach age *x* and stage *j*. These curves revealed that exposure to the LC_5_ and LC_10_ of thiamethoxam enhanced the survival rates across the developmental and adult stages. The *l_x_*, *m_x_*, and *l_x_m_x_* parameters of *R. padi* were notably affected at the LC_5_ and LC_10_ of thiamethoxam compared to untreated group (Figure 1). The *e_xj_* curves exhibit the expected survival duration for an individual *R. padi* at age *x* and stage *j* to survive after age *x* (Figure S2). Notably, the F_1_ generation of *R. padi* potentially experienced a longer lifespan when the parental generation (F_0_) was exposed to the LC_5_ and LC_10_ of thiamethoxam compared to the control. Figure S3 shows the reproductive value (*v_xj_*), indicating the population’s adherence to potential offspring from age *x* and stage *j*.

### 2.5. Population Projection

**Figure 2:**
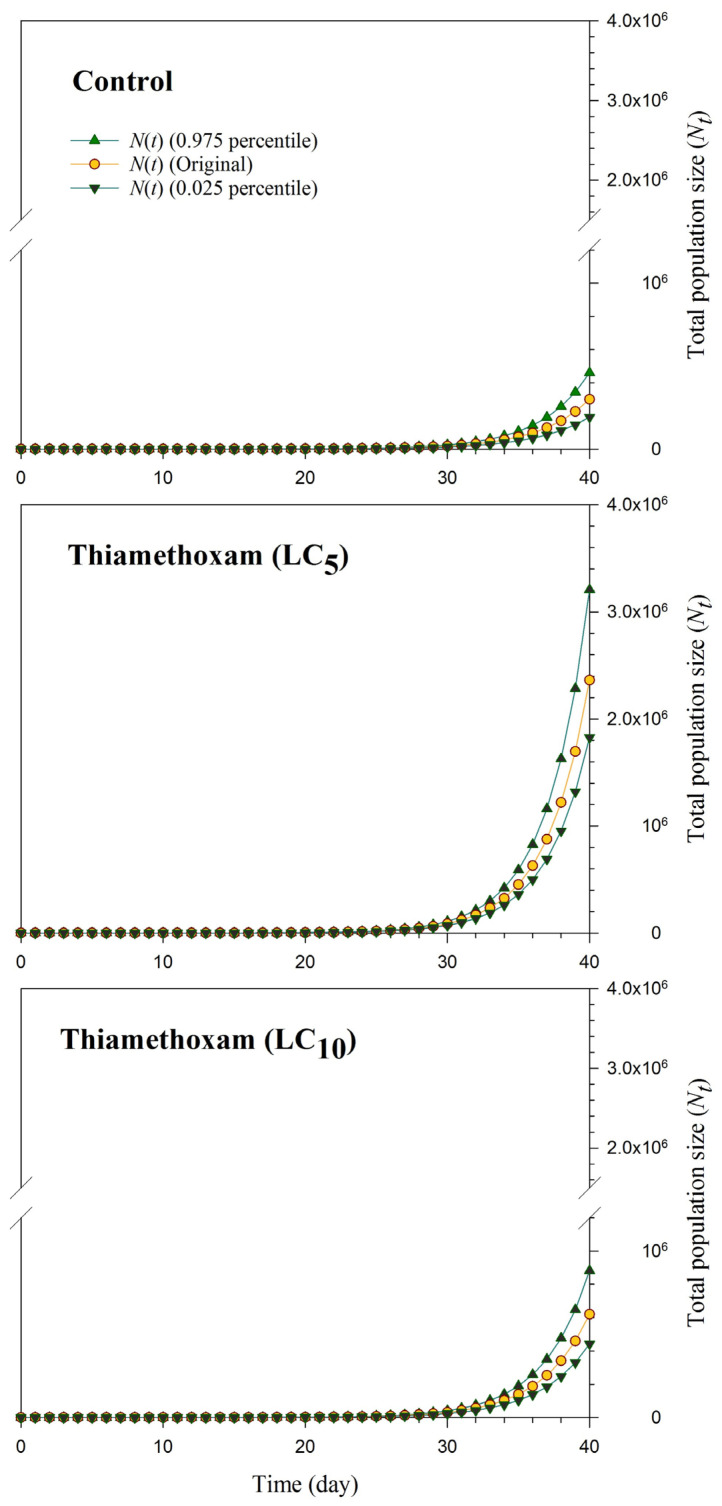
The total population size (*N_t_*) for control and F_1_ progeny of *Rhopalosiphum padi*, treated with LC_5_ and LC_10_ of thiamethoxam, was projected over 40 days by using the life table data from the original cohort and cohorts based on the 2.5 and 97.5% percentiles of *R*_0_.

The population projection (original, 2.5th, and 97.5th percentiles) for the progeny generation (F_1_) of *R. padi*, whose parents were exposed to LC_5_ and LC_10_ thiamethoxam exposures displayed in Figure 2. The projected *R. padi* population size varied, and the control group yielded the lowest population size, with approximately 300,000 individuals. In contrast, the LC5 thiamethoxam treatment resulted in the highest population size, exceeding 2,360,000 individuals. Subsequently, the LC10 thiamethoxam treatment produced nearly 620,000 individuals after 40 days (Figure 2).

### 2.6. Sublethal Effects of Thiamethoxam on Feeding Behavior of F_0_ and F_1_ Rhopalosiphum padi

The impact of sublethal concentrations of thiamethoxam significantly affect the feeding behavior of the parental generation *R. padi*, in comparison to the control (Table 4). Specifically, the mean duration of C significantly increased (*p* < 0.05) in the LC_10_ thiamethoxam exposure (8188.7 s) compared to LC_5_ (5381.4 s) and the control (4112.7 s). Conversely, the total duration of E2 notably decreased (*p* < 0.05) in both LC_10_ (7112 s) and LC_5_ (10,241 s) treatments when compared to untreated aphids (14,261 s). However, durations of Np increased significantly (*p* < 0.05) at the LC_5_ of thiamethoxam, while G and E1 showed no significant effects (*p* > 0.05).

Table 5 explores the intergenerational sublethal effects of thiamethoxam concentrations (LC_5_ and LC_10_) on the feeding behavior of F_1_ adult *R. padi* following the treatment of the parental aphids (F_0_). The results highlighted significant differences: the total duration of C in LC_5_ treatment (2315.5 s) was significantly (*p* < 0.01) shorter followed by LC_10_ (2496.4 s) as compared to the control (5269.5 s). Additionally, the total duration of E2 significantly (*p* < 0.01) decreased in both LC_5_ (15,711 s) and LC_10_ (15,326 s) of the thiamethoxam treatments compared to untreated individuals (11,287 s). Furthermore, the Np, G, and E1 durations displayed no significant effects (*p* > 0.05) following exposure to sublethal thiamethoxam concentrations.

## 3. Material and Methods

### 3.1. Toxicity Bioassays

*Rhopalosiphum padi* was initially collected from a wheat field and was reared on wheat plants under laboratory conditions (18 °C, 60 ± 5% RH, and 16:8 L: D photoperiod) for more than 2 years without exposure to any insecticide. The bioassays were conducted to assess the toxicity of thiamethoxam against adult *R. padi*. The insecticide was diluted into five tested concentrations using distilled water. All serial concentrations were immediately used in bioassays after preparation. The wheat plants were treated with all five concentrations individually using a hand sprayer until run-off (adaxial and abaxial leaf sides). Distilled water was sprayed as a control treatment. Subsequently, all treated pots were allowed to air dry at room temperature for 1–2 h. Thirty apterous adult *R. padi* were added to each pot and were considered as one replicate. Each concentration was replicated three times. The treated pots containing aphids were placed under controlled conditions (18 °C, 60 ± 5% RH, and 16:8 L: D photoperiod). The mortality was evaluated after 48 h of thiamethoxam exposure. If the aphids did not move after being lightly touched with a soft brush, they were assumed to be dead.

### 3.2. Sublethal Effects of Thiamethoxam on Rhopalosiphum padi (F_0_)

The sublethal effects of thiamethoxam on directly exposed *R. padi* (F_0_) were examined using the LC_5_ (2.844 mg L^−1^) and LC_10_ (3.869 mg L^−1^) concentrations. The LC_5_ and LC_10_ of thiamethoxam were sprinkled by a hand sprayer to the healthy wheat plants till run-off occurred (adaxial and abaxial leaf sides). Wheat plants were sprayed with distilled water as a control treatment. The treated wheat plants were allowed to dry at room temperature for 1 to 2 h. About 200–300 apterous adult aphids were reared on thiamethoxam-treated wheat plants and the control under laboratory conditions (18 °C, 60 ± 5% RH, and 16:8 L:D photoperiod). Forty healthy aphids that had survived after 48 h of exposure were selected from each treatment (water, LC_5_, or LC_10_) and were transferred to a micro cage containing non-treated fresh wheat plants. Each individual aphid was treated as a single replicate across all groups. Daily monitoring of fertility and longevity were observed until all aphids died. After being counted, newly hatched nymphs were collected from their designated cages.

### 3.3. Intergenerational Impact of Thiamethoxam on Rhopalosiphum padi (F_1_)

The same experimental technique was used to investigate the intergenerational effect of LC_5_ and LC_10_ of thiamethoxam on the subsequent progeny generation (F_1_) of *R. padi*. The 40 newly emerged nymphs from the F_0_ generation were chosen from each treatment (water, LC_5_, or LC_10_) and placed in sanitized micro cages containing fresh wheat plants, ensuring the absence of insecticide residue. Each individual wheat aphid of *R. padi* was treated as a single replicate across all groups. Survival and developmental duration of F_1_ aphids were observed daily and nymphs per aphid were noted until all aphids died. The nymphs were taken out of their cages after being counted. The experiments were carried out under standard laboratory conditions, as previously described.

### 3.4. Electro Penetrography of Rhopalosiphum padi Feeding Behavior

Feeding behavior of *R. padi* was investigated using an eight-channel DC-EPG (Wageningen University, The Netherlands) after exposure of wheat plants to the LC_5_ and LC_10_ of thiamethoxam. Additionally, the intergenerational effects of thiamethoxam were also investigated on the feeding behavior of the adult offspring generation (F_1_). The recording was carried out in accordance with the manufacturer’s recommendations. A hand sprayer was used to apply the LC_5_ and LC_10_ of thiamethoxam to the wheat plants until run-off occurred on the upper (adaxial) and lower (abaxial) leaf surfaces. As a control, plants were sprayed with distilled water. Prior to starting the feeding trials, all of the treated plants were allowed to dry in the open air for 2 h at room temperature. Adult *R. padi* were starved for about an hour between the wiring and the commencement of the EPG experiment. After starving, a small droplet of high-purity silver conductive paint was employed to attach a gold wire (18 m in diameter, 6–8 cm in length) onto the dorsum of the aphids. Subsequently, these aphids were delicately positioned on treated-wheat plants using gold wires which were then connected to a Giga-8 DC-EPG amplifier possessing 10^9^ Ω input resistance and an adjustable plant voltage. To generate voltage, copper wire (5 cm in length and 2 mm in diameter) served as a plant electrode, inserted into the soil of the pot.

Waveforms were continuously recorded from eight plants with alternate channels of water or thiamethoxam-treated plants. The experiments were conducted in an electrically earthed Faraday cage (2 × 2 × 4 feet, aluminum frame with a steel base) to avoid the electrical noises. The experiments were performed under 18 °C and 60–65% RH with continuous light for eight hours using PROBE 3.4 software (Wageningen Agricultural University, Wageningen, The Netherlands) operating in Windows-XP. Fresh wheat plants containing aphids were used for each replication. The EPGs were recorded for 8 h for each treatment. The Stylet+ Software was used to analyze the EPG recordings and the variables were processed using EPG-Excel Data Workbook according to EPG ParProc. The EPG waveforms associated with the probing activity of aphids were described as Np: total duration of non-probing, C: total duration of intercellular stylet pathway, G: total duration of xylem ingestion, E1: total duration of salivary secretion into the sieve element, and E2: total duration of phloem sap ingestion and concurrent salivation.

### 3.5. Data Analysis

LC_5_, LC_10_, and LC_50_ values of thiamethoxam against *R. padi* were calculated using the log-probit model through PoloPlus 2.0 (LeOra Software Inc., Berkeley, CA, USA). The feeding behavior data was subjected to statistical analysis using a one-way analysis of variance (ANOVA) with Tukey’s post hoc test in IBM SPSS Statistics 22.0 (IBM, Chicago, IL, USA).

### 3.6. Life Table Data Analysis

The age-stage, two-sex life table method was used to evaluate the life table data of thiamethoxam-treated parental aphids (F_0_) and their offspring (F_1_) [25]. The demographic traits such as intrinsic rate of increase (*r*), finite rate of increase (*λ*), mean generation time (*T*), and net reproductive rate (*R*_0_), and the life-history traits were determined using the computer program TWOSEX-MS Chart [31]. The 100,000 bootstrap replicates were employed to compute differences and standard errors (SEs) [32,33,34,35]. Based on the confidence interval of difference, a paired bootstrap test was conducted to examine demographic parameter variations among the control, LC_5_, and LC_10_ treated groups at a 5% significance level [36].

### 3.7. Population Projection

The TIMING-MSChart program [37] was utilized to develop population projections for control, LC5, and LC10 cohorts, employing a methodology based on life table parameters [38]. The projections spanned 40 days, assuming conditions without the suppression of both biotic and abiotic factors. Each cohort commenced with 10 nymphs. Employing a bootstrap approach, 100,000 bootstrap results of the net reproductive rate (*R*_0_) were arranged, and percentiles at 2.5 and 97.5 were computed, corresponding to the 2500th and 97,500th samples from the sorted data. Life table datasets generated via bootstrapping, representing the 2.5 and 97.5 percentiles of *R*_0_, were then used to project population dynamics for another 40 days, illustrating variation and uncertainty through confidence intervals [39].

## 4. Discussion

The age-stage, two-sex life tables approach and electrical Penetration Graphs (EPGs) were used to explore the intergenerational sublethal effects of thiamethoxam on the demographic parameters and feeding behavior of *R. padi* (F_0_ and F_1_). Bioassay results showed that thiamethoxam exhibited high toxicity against adult *R. padi* with an LC_50_ of 11.458 mgL^−1^ after 48 h treatment. Upon degradation, insecticides at sublethal concentrations exert a profound influence on the behavioral and physiological traits of exposed individuals as well as inducing resistance development [40,41,42]. These results suggest that the LC_5_ and LC_10_ of thiamethoxam might be crucial to study how these concentrations affect the biological parameters and feeding behavior for effectively managing *R. padi*.

The results showed that sublethal concentrations of thiamethoxam notably decreased the lifespan, fertility, and reproductive span of adult *R. padi* (F_0_) when directly exposed. Our findings are consistent with previous studies that the fertility and longevity of *A. gossypii* and *S. graminum* was dramatically decreased after exposure to sublethal concentrations of thiamethoxam and flonicamid [42,43]. Likewise, reduced fecundity and longevity were observed in different insects following exposure to the sublethal concentrations of insecticides [44,45,46]. Our findings align with previous studies indicating a significant reduction in the fecundity of *A. gosspyii* when exposed to sublethal and low-lethal concentrations of flonicamid [47]. Flupyradifurone sublethal concentrations exposed to *M. persicae* were reported to reduce longevity and reproductive capabilities [48]. Hence, the results highlight that beyond their lethal impact, sublethal concentrations of insecticides also have a detrimental effect on the longevity and fecundity of surviving aphids.

The intergenerational effects of thiamethoxam exhibited substantial alterations on the fecundity and demographic parameters of offspring generations of *R. padi* (F_1_). The findings showed that at LC_5_ of thiamethoxam, the developmental durations of 1st, 3rd, and 4th instar of F_1_ *R. padi* were significantly reduced; however, the LC_10_ group showed no significant effects when compared to the control. Moreover, the duration of 2nd-instar nymph was significantly decreased at both concentrations of thiamethoxam. The sublethal concentrations (LC_10_ and LC_25_) of broflanilide considerably prolonged the developmental duration of 3rd-instar *M. persicae*. Additionally, the LC_25_ treatment resulted in a notable reduction in the longevity and fecundity of these aphids when compared to the untreated group [20]. After being exposed to sublethal concentrations of insecticide, the developmental duration of the 1st-instar aphid, *A. gosspyii* was shown to be significantly increased [47]. However, the pre-adult stage of *R. padi* (F_1_) was significantly decreased in the offspring aphid (F_1_) compared to the control group. The LC_5_ and LC_10_ of thiamethoxam resulted in a considerable increase in adult longevity as compared to the control, while the total longevity of female progeny aphids (F_1_) was prolonged only at LC_5_. These results suggest that *R. padi* development and the total longevity were positively affected following 48 h exposure of sublethal concentrations of thiamethoxam. The fecundity (nymphs/female) and reproductive days (RP*_d_*) of F_1_ individuals were substantially increased, while no effects were observed on the adult pre-reproductive period (APRP). However, the total pre-reproductive period (TPRP) was significantly decreased at the LC_5_ and LC_10_ of thiamethoxam compared to control aphids. Gong et al. (2023) reported increased longevity and fecundity in *Nilaparvata lugens* (Stål) (Hemiptera: Delphacidae) when exposed to the LC_20_ concentration of nitenpyram [49]. In addition, the LC_5_ of thiamethoxam showed a substantial increase in key demographic characteristics of the offspring generation (F_1_) including *R*_0_, *r*, and *λ*. In contrast, no significant effects were recorded at LC_10_ as compared to the control group. The changes in the life-history characteristics of *R. padi* indicate the development of intergenerational hormetic effects resulting from the exposure of parental aphids to sublethal concentrations of thiamethoxam. *Rhopalosiphum padi* demonstrated this hormetic response without apparent fitness trade-offs after being exposed to the LC_5_ and LC_10_ concentrations of thiamethoxam. Similar hormetic effects in different traits have been observed in *A. gosspyii* [43], *M. persicae* [48,50,51], and *N. lugens* when subjected to insecticides [49]. In comparison to the control group, the LC_5_ and LC_10_ of thiamethoxam-treated groups had a significant decrease in the mean generation time and pre-adult developmental duration of *A. gossypii* [43]. The hormesis might be crucial for insect pests to withstand successive sublethal stresses [52,53]. These findings indicated that the sublethal concentrations of thiamethoxam induces intergenerational hormetic effects that ultimately causes population expansion of this key pest.

EPG monitoring of probing behavior exhibited a minimal effect of thiamethoxam on piercing-sucking herbivores [54]. The effects of thiamethoxam sublethal concentrations (LC_5_ and LC_10_) on the feeding pattern of both parental and progeny *R. padi* were also investigated in this context by using electric penetration graph recordings (EPG). The findings also indicated a notable decrease in the total duration of E2 among F_0_ adults exposed to sublethal concentrations of thiamethoxam, while the C waveforms notably increased at the LC_10_ concentration of thiamethoxam. In contrast, the E2 waveforms dramatically enhanced at LC_5_ followed by LC_10_ and the control group of F_1_ aphids. However, thiamethoxam at the LC_5_ and LC_10_ concentrations decreased the total duration of C as compared to control. These results are consistent with previous studies that the phloem sap ingestion phase was notably reduced in *S. germanium*, *A. gossypii* and *Sitobion avenae* (Fabricius) (Hemiptera: Aphididae) when exposed to sublethal concentrations of flonicamid, cycloxaprid, thiamethoxam, imidacloprid, dinotefuran and thiacloprid [42,55,56]. The ingestion phases of the cotton leafhopper, *Amrasca biguttula* (Ishida) (Hemiptera: Cicadellidae) were significantly inhibited with the increasing concentration of flonicamid [57]. Imidacloprid and cyantraniliprole at sublethal concentrations notably extended the encountered mechanical probing difficulties (F) and the total durations of the intercellular stylet pathway (C) in *M. persicae* when feeding on treated tobacco plants [58]. Meanwhile, increasing concentration of flonicamid and imidacloprid resulted in a significant decrease in E1 and sap-feeding durations (E2) in *S. graminum* and *A. gossypii* [42,59]. All in all, these findings indicated that the LC_5_ and LC_10_ of thiamethoxam negatively affect the feeding behavior of directly treated aphids. Interestingly, thiamethoxam-induced hormetic effects were observed on the progeny generation following exposure of parental aphids to the sublethal concentrations that might be translated to the population expansion of *R. padi*.

## 5. Conclusions

To conclude, the current study demonstrated that thiamethoxam exhibited high toxicity and affects the biological parameters of directly exposed *R. padi* (F_0_). However, the sublethal concentrations of thiamethoxam (LC_5_ and LC_10_) induces intergenerational hormetic effects on the key demographic traits and feeding behavior of the progeny generation (F_1_) that might result in pest resurgence. These findings provided in-depth knowledge about the thiamethoxam-induced hormetic effect on the life-history traits as well as feeding behavior of this economically important pest that might be crucial in understanding how to tackle the pest resurgence issue. However, future studies are needed to validate these findings under field contexts as well as to investigate the underlying molecular mechanisms of insecticide-induced hormesis.

## Figures and Tables

**Table 1:** Sublethal effects of thiamethoxam LC_5_ and LC_10_ on adult longevity, fecundity, and reproductive days of the F_0_ generation of *Rhopalosiphum padi* (Mean ± SE).

Parameters	Control	Thiamethoxam (LC_5_)	Thiamethoxam (LC_10_)
	Mean ± SE	Mean ± SE	Mean ± SE
Adult longevity (days)	25.05 ± 0.12 ^a^	19.00 ± 0.29 ^b^	14.00 ± 0.32 ^c^
Fecundity (nymphs/female)	48.00 ± 0.70 ^a^	41.00 ± 0.84 ^b^	32.00 ± 0.63 ^c^
Reproductive days (days)	22.73 ± 0.25 ^a^	18.70 ± 0.33 ^b^	13.58 ± 0.27 ^c^

Bootstrap technique with 100,000 resampling was used to calculate the standard errors and paired bootstrap test (*p* < 0.05) was employed to compare the differences among different groups. Different lowercase letters within a row indicated significant differences among the treatments.

**Table 2:** Duration (days) of different developmental stages of *Rhopalosiphum padi* F_1_ generation, descended from parents (F_0_) treated with LC_5_ and LC_10_ of thiamethoxam (Mean ± SE).

Stage		Control		Thiamethoxam (LC_5_)		Thiamethoxam (LC_10_)
	*n*	Mean ± SE	*n*	Mean ± SE	*n*	Mean ± SE
First-instar nymph	40	1.45 ± 0.09 ^a^	40	1.20 ± 0.06 ^b^	40	1.33 ± 0.07 ^ab^
Second-instar nymph	38	1.24 ± 0.07 ^a^	40	1.03 ± 0.02 ^b^	39	1.03 ± 0.03 ^b^
Third-instar nymph	38	1.32 ± 0.08 ^a^	38	1.11 ± 0.05 ^b^	37	1.24 ± 0.08 ^ab^
Fourth-instar nymph	37	1.41 ± 0.08 ^a^	38	1.11 ± 0.05 ^b^	35	1.20 ± 0.07 ^ab^
Pre-adult	37	5.38 ± 0.08 ^a^	38	4.42 ± 0.09 ^c^	35	4.80 ± 0.12 ^b^
Adult (Female)	37	24.00 ± 0.20 ^b^	38	27.00 ± 0.20 ^a^	35	26.00 ± 0.73 ^a^
Total longevity (Female)	37	29.38 ± 0.23 ^b^	38	31.42 ± 0.24 ^a^	35	30.80 ± 0.74 ^ab^

Bootstrap technique with 100,000 resampling was used to calculate the standard errors and paired bootstrap test (*p* < 0.05) was employed to compare the differences among different groups. Different lowercase letters within a row indicated significant differences among the treatments.

**Table 3:** Reproduction and life table parameters of *Rhopalosiphum padi* F_1_ generation, descended from parents (F_0_) treated with LC_5_ and LC_10_ of thiamethoxam (Mean ± SE).

Parameters ^a^	Control	Thiamethoxam (LC_5_)	Thiamethoxam (LC_10_)
	Mean ± SE	Mean ± SE	Mean ± SE
*R*_0_ (offspring/individual)	42.55 ± 2.01 ^b^	55.10 ± 2.10 ^a^	48.13 ± 3.18 ^ab^
*r* (day^−1^)	0.2810 ± 0.0055 ^b^	0.3311 ± 0.0065 ^a^	0.2988 ± 0.0083 ^b^
*λ* (day^−1^)	1.3244 ± 0.0073 ^b^	1.3926 ± 0.0090 ^a^	1.3482 ± 0.0112 ^b^
*T* (days)	13.35 ± 0.16 ^a^	12.11 ± 0.18 ^b^	12.96 ± 0.22 ^a^
*F* (nymphs/female)	46.00 ± 0.65 ^b^	58.00 ± 0.67 ^a^	55.00 ± 1.58 ^a^
RP*_d_*(days)	22.76 ± 0.26 ^b^	26.08 ± 0.19 ^a^	24.77 ± 0.71 ^a^
APRP (days)	0.14 ± 0.07 ^a^	0.08 ± 0.04 ^a^	0.20 ± 0.09 ^a^
TPRP (days)	5.51 ± 0.11 ^a^	4.50 ± 0.10 ^c^	5.00 ± 0.16 ^b^

Bootstrap technique with 100,000 resampling was used to calculate the standard errors and paired bootstrap test (*p* < 0.05) was employed to compare the differences among different groups. Different lowercase letters within a row indicated significant differences among the treatments. ^a^ (*R*_0_) net reproductive rate, (*r*) intrinsic rate of increase, (*λ*) finite rate of increase, (*T*) mean generation time, (*F*) fecundity, (RP*_d_*) reproductive days, (APRP) adult prereproductive period, and (TPRP) total prereproductive period.

**Table 4:** Sublethal effects of thiamethoxam on the probing and feeding behavior of *Rhopalosiphum padi* on wheat plants after treatment with the LC_5_ and LC_10_ concentrations.

Treatments	Np	C	G	E1	E2
Control	972.6 ± 279.36 ^b^	4112.7 ± 660.94 ^b^	1374.1 ± 603.65 ^a^	476.6 ± 177.69 ^a^	14,261 ± 1091.98 ^a^
LC_5_	3081.5 ± 331.49 ^a^	5381.4 ± 448.28 ^b^	1368.1 ± 392.78 ^a^	1005.3 ± 148.62 ^a^	10,241 ± 687.65 ^b^
LC_10_	2257.5 ± 619.97 ^ab^	8188.7 ± 967.94 ^a^	1258.7 ± 730.75 ^a^	959.4 ± 201.42 ^a^	7112 ± 487.52 ^c^

Np (total duration of non-probing); (C) total duration of the intercellular stylet pathway, (G) total duration of xylem ingestion, (E1) total duration of salivary secretion into the sieve element, (E2) total duration of phloem sap ingestion and concurrent salivation. The letters within the same column indicated significant differences at *p* < 0.05 level (one-way ANOVA followed by Tukey’s post hoc test).

**Table 5:** Intergenerational effects of thiamethoxam on the probing and feeding behavior of progeny *Rhopalosiphum padi* (F_1_) whose parents (F_0_) were treated with the LC_5_ and LC_10_ concentrations.

Treatments	Np	C	G	E1	E2
Control	1358.7 ± 373.08 ^a^	5269.5 ± 829.03 ^a^	2932.7 ± 1226.44 ^a^	342.33 ± 87.66 ^a^	11,287 ± 1708.69 ^b^
LC_5_	1240.3 ± 261.34 ^a^	2315.5 ± 389.59 ^b^	699.7 ± 335.34 ^a^	684.3 ± 125.59 ^a^	15,711 ± 583.21 ^a^
LC_10_	2061.4 ± 403.84 ^a^	2496.4 ± 353.60 ^b^	998.2 ± 494.17 ^a^	670.38 ± 123.40 ^a^	15,326 ± 713.29 ^a^

Np (total duration of non-probing); (C) total duration of the intercellular stylet pathway, (G) total duration of xylem ingestion, (E1) total duration of salivary secretion into the sieve element, and (E2) total duration of phloem sap ingestion and concurrent salivation. The letters within the same column indicated significant differences at *p* < 0.05 level (one-way ANOVA followed by Tukey’s post hoc test).

## Data Availability

All data analyzed during this study are included in this published article.

## Supplementary Materials

The supporting information including Table S1. Toxicity of thiamethoxam against adult *Rhopalosiphum padi* after 48 h exposure, Figure S1. Age-stage specific survival rate (*s_xj_*) of F_1_ generation *Rhopalosiphum padi* produced from F_0_ individuals treated with the sublethal concentrations of thiamethoxam, Figure S2. Age-stage life expectancy (*e_xj_*) of F_1_ *Rhopalosiphum padi* originated from F_0_ aphids treated with the sublethal concentrations of thiamethoxam, Figure S3. Age-stage reproductive value (*v_xj_*) of progeny generation *Rhopalosiphum padi* (F_1_) originated from F_0_ aphids treated with the sublethal concentrations of thiamethoxam can be downloaded at: https://www.mdpi.com/article/10.3390/plants13060865/s1.
