# Supplementary material for: Thiamethoxam-Induced Intergenerational Sublethal Effects on the Life History and Feeding Behavior of Rhopalosiphum padi
Source: Plants (Basel). 2024 Mar 17;13(6):865. doi: 10.3390/plants13060865 (PMC10975832; doi:10.3390/plants13060865)
Supplement: Supplementary file 1 [file plants-13-00865-s001.zip › plants-2886892-supplementary.pdf]

Supplementary file

Table S1. Toxicity of thiamethoxam against adult *Rhopalosiphum padi* after 48 h exposure.

**Treatments**	**Slope ± SE**[a]	**LC_5 mg/l (95% CL)**[b]	**LC_{10} mg/l (95% CL)**[b]	**LC_{50} mg/l (95% CL)**[b]	**$\chi 2$ (*df*)**[c]	***P*-value**
Thiamethoxam	2.718 ± 0.283	2.844 (1.900-3.762)	3.869 (2.772-4.902)	11.458 (9.766-13.416)	5.458 (13)	0.963

[a] Standard error.

[b] 95% confidence intervals.

[c] Chi-square value ($\chi 2$) and degrees of freedom (*df*) calculated by PoloPlus 2.0.

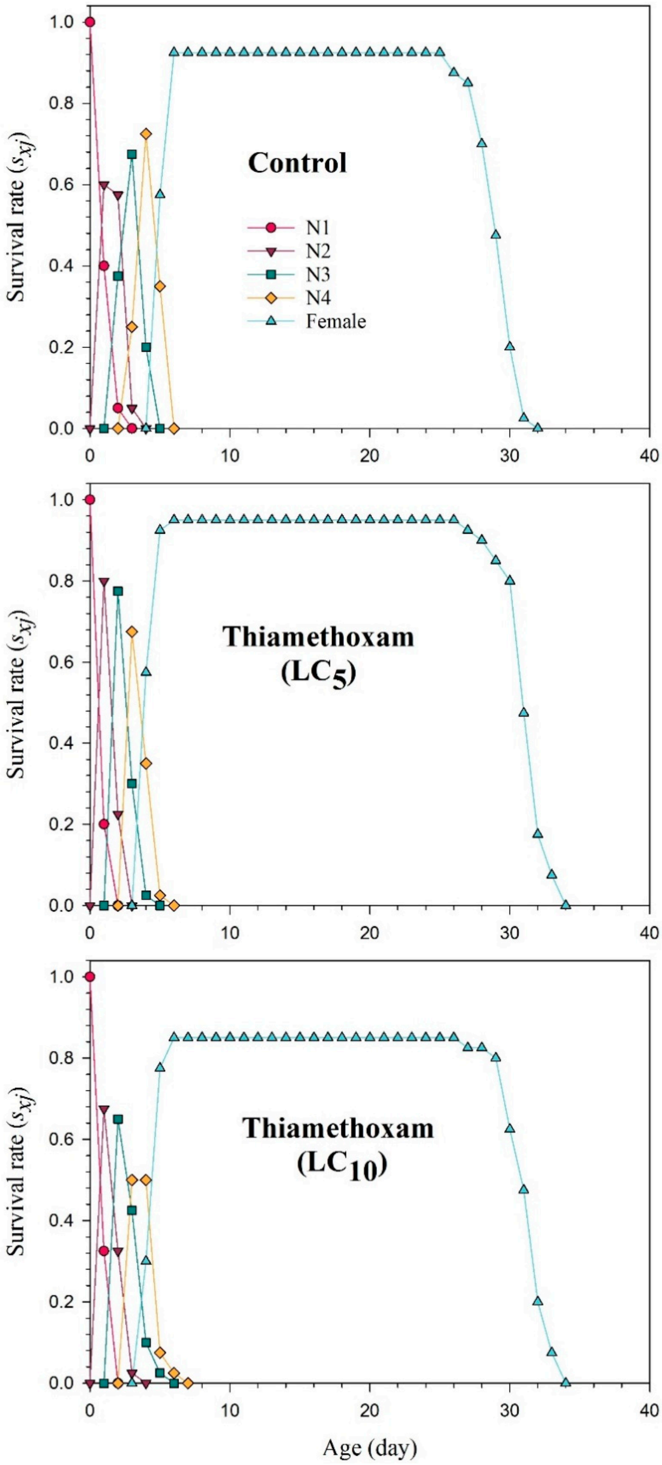

Figure S1. Age-stage specific survival rate (s_{xj}) of F_1 generation *Rhopalosiphum padi* produced from F_0 individuals treated with the sublethal concentrations of thiamethoxam. N1: first-instar nymph, N2: second-instar nymph, N3: third-instar nymph, and N4: fourth-instar nymph.

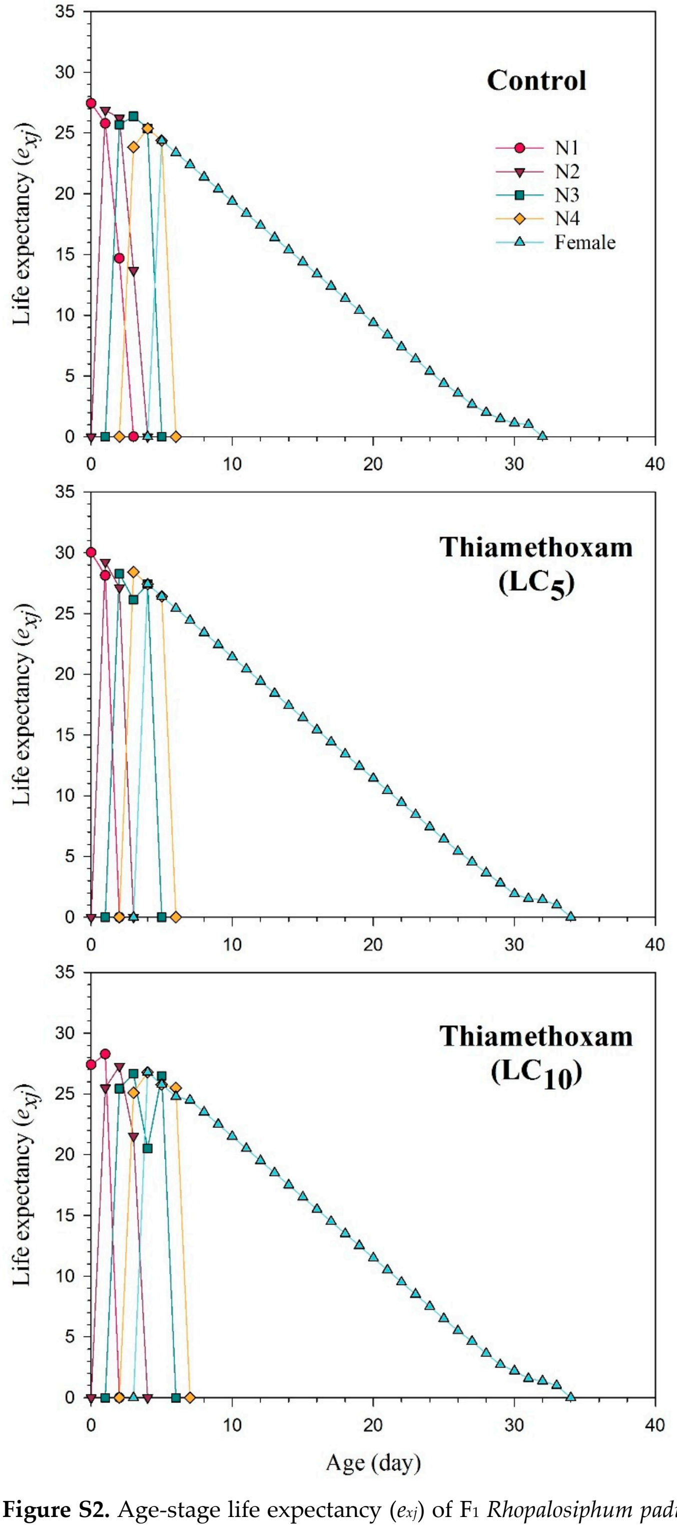

Figure S2. Age-stage life expectancy (e_{xj}) of F_1 *Rhopalosiphum padi* originated from F_0 aphids treated with the sublethal concentrations of thiamethoxam. N1: first-instar nymph, N2: second-instar nymph, N3: third-instar nymph, and N4: fourth-instar nymph.

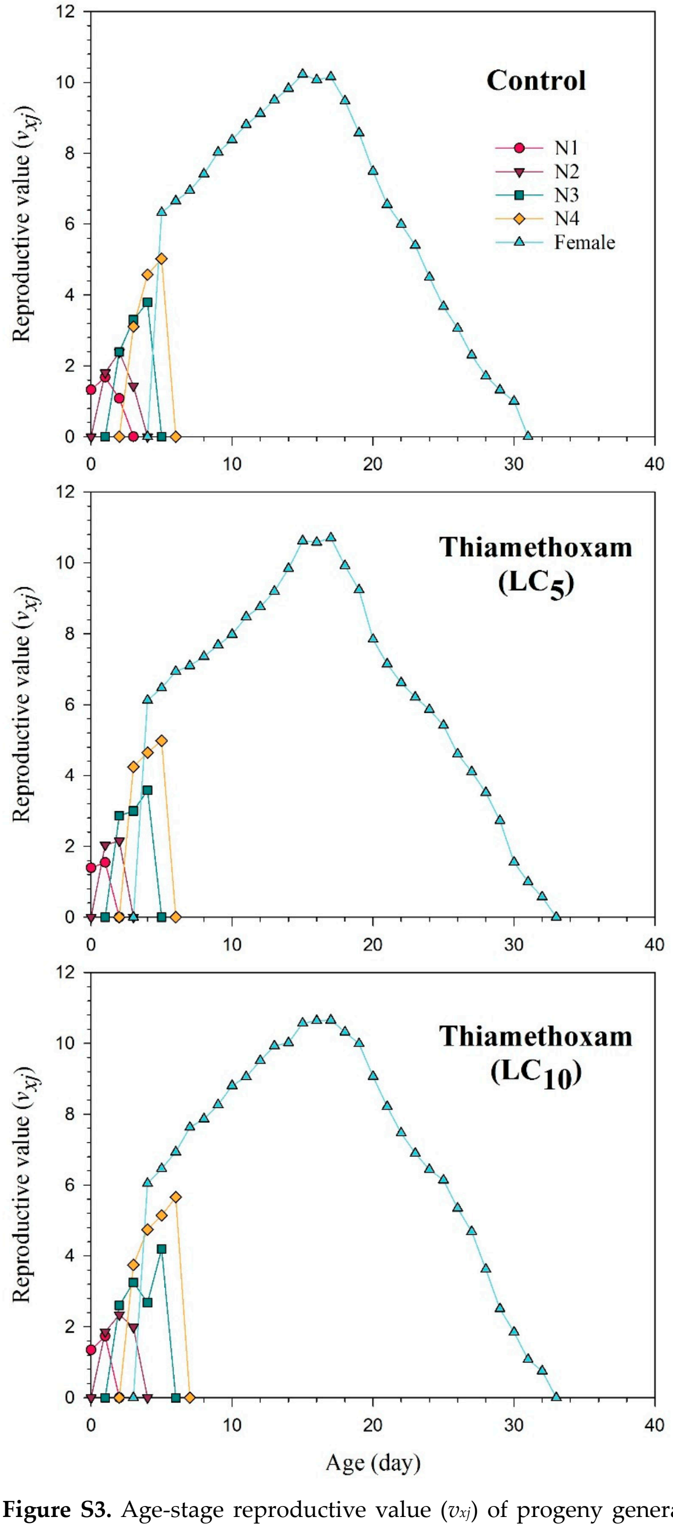

Figure S3. Age-stage reproductive value (v_{xj}) of progeny generation *Rhopalosiphum padi* (F_1) originated from F_0 aphids treated with the sublethal concentrations of thiamethoxam. N1: first-instar nymph, N2: second-instar nymph, N3: third-instar nymph, and N4: fourth-instar nymph.
